# Association between physical activity dimensions and the risk of hypertension among middle and older adults: A cross-sectional study in China

**DOI:** 10.3389/fpubh.2022.995755

**Published:** 2022-09-24

**Authors:** Linlin Zhou, Wei Feng, Na Xiang, Yue Cheng, Xudong Ya, Mingxia Wang, Xingqi Wang, Yujia Liu

**Affiliations:** ^1^Institute of Physical Education, Jiangsu Normal University, Xuzhou, China; ^2^Department of Physical Education, Suzhou University, Suzhou, China; ^3^Caoxian People's Hospital, Heze, China; ^4^School of Life Science, Biomedical R&D Center, Jiangsu Normal University, Xuzhou, China

**Keywords:** hypertension risk, middle and older adults, restricted cubic spline, dose-response relationship, physical activity

## Abstract

**Background:**

It is known that insufficient physical activity is associated with the risk of hypertension, but the relationship to different physical activity dimensions within hypertension risk remains to be elucidated.

**Objective:**

The objective of this study is to identify the association between physical activity intensity, frequency, duration, and volume with hypertension risk. Meanwhile, a dose-response experiment is conducted to determine the relationship between physical activity level and hypertension risk.

**Methods:**

Data came from the 2018 China Health and Retirement Longitudinal Study (CHARLS, 2018), which included 14266 participants over the age of 45. Binary logistic regression models were established to assess the associations between different dimensions of physical activity and the risk of hypertension. Restricted cubic spline analysis was used to examine possible non-linear associations between physical activity volume and hypertension risk.

**Results:**

For frequency, lower hypertension risk was associated with performing vigorous physical activity 6–7d/w (OR 0.82, 95%CI 0.73–0.93) and moderate physical activity 6–7d/w (OR 0.89, 95%CI 0.80–0.99). No significant association between any light physical activity frequency and hypertension was observed before and after being adjusted. For the duration, lower hypertension risk was observed in performing vigorous physical activity ≥240 min/d (OR 0.85, 95%CI 0.75–0.97) and moderate physical activity ≥240 min/d (OR 0.83, 95%CI 0.71–0.97). For volume, the risks of hypertension in the participants who reported TPA in the 3th and 4th of quantiles were reduced by 18% (OR 0.82, 95%CI 0.72–0.95) and 22% (OR 0.78, 95%CI 0.68–0.91). A non-linear dose-response association between total physical activity and the risk of hypertension was shown among all of the participants (P non-linearity < 0.05).

**Conclusion:**

Higher frequency and longer duration of vigorous physical activity or moderate physical activity were significantly associated with a lower risk of hypertension. Higher physical activity levels were associated with a lower risk of hypertension and there was an inverse non-linear dose-response relationship between weekly total physical activity and the risk of hypertension. These findings provide further proof that hypertension could be prevented through increased physical activity.

## Introduction

Hypertension is one of the most common chronic diseases worldwide (1), and it is the leading risk factor for cardiovascular disease (2). Hypertension can lead to serious health problems such as heart disease, stroke, as well as possible death if left uncontrolled (3). About 1 billion people are living with hypertension worldwide (4), and more than 1.56 billion people will have elevated hypertension in 2025 (1, 5). Likewise, hypertension is common in China. A large-scale epidemiological survey in 2013 showed that the prevalence of hypertension among Chinese adults was as high as 27.8% (6). Studies have shown that many factors are related to the risk of hypertension such as obesity, alcohol consumption, sodium intake, and physical inactivity (7–10). However, previous data showed that more than 40% of the elderly in China are still physically inactive (11). In addition, the prevalence of hypertension increases with age (12). Promoting adequate physical activity in middle-aged and older adults to prevent hypertension is a key issue.

Increased physical activity (PA) was effective in the treatment and prevention of hypertension, this viewpoint has been confirmed by many studies (13–15). An inverse dose-response association between levels of PA and hypertension was demonstrated in a meta-analysis (16). But this association may vary according to sex and age. An early study suggested that increased total amount of energy expenditure during PA was statistically associated with reduced risk of hypertension among men but not among women (17). Additionally, Cohen et al. (18) found age differences in the association between PA and hypertension, the association between high levels of PA and the prevention of hypertension was weaker among older women when compared to younger women. What's more, there were also inconsistent results regarding the association between the intensity of PA and the risk of hypertension. It was reported that vigorous physical activity (VPA) is associated with a reduced risk of hypertension, and VPA was far more effective than moderate physical activity (MPA) or light physical activity (LPA) in the prevention of hypertension (19). An earlier study, however, found that the addition of VPA cannot offer additional benefits in the prevention of hypertension (20). The optimal prescription (intensity, duration, frequency and volume) for the prevention and treatment of hypertension remains elusive.

Further investigation of the association between physical activity to hypertension is still required. We hypothesized that higher intensity, frequency, duration and volume of physical activity would each be associated with reduced hypertension risk. Therefore, this study aimed to assess the association of hypertension with various dimensions of PA and to assess whether there were gender and age differences in the dose-response relationship between PA and the risk of developing hypertension.

## Materials and methods

### Participants

This cross-sectional study was done based on the China Health and Retirement Longitudinal Study (CHARLS) survey in 2018. CHARLS is a nationally representative survey of Chinese middle-aged and elderly people conducted by the National School for Development (China Center for Economic Research) in collaboration with Peking University's Institute for Social Science Survey. The survey covers 450 urban communities and rural areas in 28 provinces, municipalities, and autonomous regions in China. It was approved by the Biomedical Ethics Review Committee of Peking University (approval number: IRB00001052-11015), and informed consent was required of all participants.

To be included in this study, participants (age ≥ 45 years) should have complete data on physical activity record, gender, place of residence, marital status, educational level, sleep status, smoking status, drinking status, annual income, hypertension record. Participants younger than 45 years or with missing data for any of the above will be excluded.

Among 19,752 participants in 2018, we excluded 226 participants aged < 45 years, 5,243 participants with missing hypertension diagnoses data, 12 participants with missing sleep data, and 5 participants with missing smoking data. Finally, 14,266 participants were included in this cross-sectional study. Based on the *post hoc* analysis of sample power using G Power 3.1, the present study population provided a statistical power of 0.99.

### Physical activity

A modified version of the validated International Physical Activity Questionnaire Short Form (IPAQ-SF) (21) was used to measure participants' physical activity (see Supplementary material). Each participant was asked, “Do you usually take this type of activity for at least 10 min every week?” Specific types of physical activity include (1) VPA: activities that cause shortness of breath and may include carrying heavy stuff, digging, hoeing, aerobic workout, bicycling at a fast speed, and riding a cargo bike or motorcycle. (2) MPA: activities that make you breathe faster than usual and may include carrying light stuff, bicycling at a normal speed, mopping, Tai-Chi, and speed walking. (3) LPA: such as walking from one place to another place at a workplace or home, and taking a walk for leisure, sports, exercise, or entertainment. If participants answered “no”, they would be considered as not engaged in this type of PA during the week. If participants answered “yes”, they were further asked about the frequency and duration of each PA intensity. The frequency of PA ranged from 0–7 d/w and was divided into 4 levels: no activity (0 d/w); 1–2 d/w; 3–5 d/w; and 6–7 d/w. The duration of PA was categorized into 5 levels by CHARLS: no activity; 10–29 min/d; 30–119 min/d; 120–239 min/d; and ≥240 min/d. Metabolic equivalent of task (MET) was cited to calculate the volume of physical activity with considerations of intensity. Resting energy expenditure during quiet sitting was defined as one MET. According to the Physical Activity Guidelines Advisory Committee Scientific Report of 2018 (22), we assigned 6.0 METs, 3.1 METs, and 1.6 METs for 1 min of VPA, MPA, and LPA, respectively. The weekly PA volume for each intensity category equals the product of PA frequency, the daily duration of PA, and the value assigned for each category. The total volume of PA (TPA) equals the sum of VPA, MPA, and LPA. Finally, the TPA was classified by quartiles.

### Hypertension

Hypertension was defined based on self-reported medical history and measurement of blood pressure (systolic blood pressure ≥140 mmHg or diastolic blood pressure ≥90 mmHg). Participants were asked the question “Have you been diagnosed with hypertension by a doctor?” We considered that the individual had hypertension when the response was “yes”.

### Covariates

Covariates in the present study were age, sex (male, female), marital status (married, separated/divorced/widowed, never married), education (junior high school or less, senior high school and vocational school, college or higher), residency (urban, rural), smoking status (current smoker, quit smoking, never smoked), drinking frequency (≤1/month,>1/month, never) and sleep duration (<7 h,7–8 h, ≥8 h), annual income (<5000 yuan, 5000–10000 yuan, 10000–30000 yuan, 30000–50000 yuan, ≥50000 yuan).

### Statistical analysis

The software STATA16.0 was used for data processing and analysis. Categorical variables were expressed as counts and percentages and were compared using the Pearson's chi-squared test. Continuous data are expressed as mean ± SD and analyzed with Student's *t*-test. Binary logistic regression analysis was used to explore the associations between various dimensions of PA and the risk of hypertension, while adjusting for potential confounders. Model 1 was adjusted for demographic characteristics (including age, sex, marital status, education, residency); Model 2 was further adjusted for health-related behaviors (smoking status, drinking frequency, sleep duration). A restricted cubic spline (RCS) was used to assess the dose-response relationship between the risk of hypertension and PA, using the 25th, 50th, and 75th percentiles of the TPA as fixed knots. The RCS models were adjusted for age, sex, marital status, education, residency, smoking status, drinking frequency and sleep duration. Analyses stratified by sex and age were conducted. Missing data were imputed using multiple imputation by chained equations. To test the robustness of the results, we performed a sensitivity analysis with imputed data. All tests were two-tailed, and a probability value of *p* < 0.05 was considered statistically significant. Graph Pad prism 8 was used to draw cubic splines.

## Results

### Baseline characteristics

Table 1 showed the demographic data of participants. Among 14 266 participants in this study, 2,175 (15.25%) reported having hypertension. The risk of hypertension was significantly correlated with age (*P* < 0.001), sex (*P* = 0.006), marital status (*P* < 0.001), smoking status (*P* < 0.001) and annual income (*P* = 0.019). However, there was no significant difference in drinking frequency, residency, education, and sleep duration between participants with and without hypertension (all *P* > 0.05). Compared with non-hypertensive participants, hypertensive patients had lower weekly TPA. Women and older adults had lower weekly TPA (Supplementary Table S1).

**Table 1:** Baseline characteristics of participants.

**Characteristic**	**Total** **(*N* = 14,266)**	**Hypertension** **(*N* = 2,175)**	**Non-hypertension** **(*N* = 12,091)**	***P*** **-value**
**Age, years**, ***n*** **(%)**				<0.001
<65	9,483	1,224 (56.28)	8,259 (68.31)	
≥65	4,783	951 (43.72)	3,832 (31.69)	
**Sex**, ***n*** **(%)**				0.006
Male	6,903	1,111 (51.08)	5,792 (47.90)	
Female	7,363	1,064 (48.92)	6,299 (52.10)	
**Marital status**, ***n*** **(%)**				<0.001
Married or cohabiting	12,371	1,801 (82.80)	10,570 (87.42)	
Separated, divorced or widowed	1,808	364 (16.74)	1,444 (11.94)	
Never married	87	10 (0.46)	77 (0.64)	
**Drinking frequency**, ***n*** **(%)**				0.192
≤ 1/month	3,905	575 (26.44)	3,330 (27.54)	
>1/month	1,132	158 (7.26)	974 (8.06)	
Never	9,229	1,442 (66.30)	7,787 (64.40)	
**Smoking status**, ***n*** **(%)**				<0.001
Current smoker	4,076	585 (26.90)	3,491 (28.87)	
Quit	1,891	385 (17.70)	1,506 (12.46)	
Never	8,299	1,205 (55.40)	7,094 (58.67)	
**Residency**, ***n*** **(%)**				0.667
Urban	4,003	602 (27.68)	3,401 (28.13)	
Rural	10,263	1,573 (72.32)	8,690 (71.87)	
**Education**, ***n*** **(%)**				0.566
Junior high school or less	12,421	1,909 (87.77)	10,512 (86.94)	
Senior high school or vocational school	1,723	248 (11.40)	1,475 (12.20)	
College or higher	122	18 (0.83)	104 (0.86)	
**Sleep duration, hours**, ***n*** **(%)**				0.339
<7	7,921	1,239 (56.97)	6,682 (55.26)	
7-8	2,507	370 (17.01)	2,137 (17.67)	
≥ 8	3,838	566 (26.02)	3,272 (27.06)	
**Annual income, yuan**, ***n*** **(%)**				
< 5000	8,972	1,430 (65.75)	7,542 (62.38)	0.019
5000–30000	2,973	430 (19.77)	2,543 (21.03)	
30000–50000	1,444	199 (9.15)	1,245 (10.30)	
≥ 50000	877	116 (5.33)	761 (6.29)	
**TPA, MET-mins/week, mean (SD)**	5,122 (5629)	4,520 (5365)	5,230 (5669)	<0.001

### Logistic regression analysis of physical activity and hypertension

#### Total physical activity and the risk of hypertension

Table 2 shows the results of logistic regression analysis between TPA and the risk of hypertension. Participants who reported TPA in the 3th and 4th of quartiles had a 25% (OR 0.75, 95%CI 0.65–0.86) and 31% (OR 0.69, 95%CI 0.60–0.79) lower risk of hypertension compared to the first quartile of TPA. After adjusting for confounding factors including age, sex, marital status, education, residency, smoking status, drinking frequency, sleep duration and annual income, the risks of hypertension in the participants who reported TPA in the 3th and 4th of quantiles were reduced by 18% (OR 0.82, 95%CI 0.72–0.95) and 22% (OR 0.78, 95%CI 0.68–0.91), respectively (all *P* < 0.01). The reduction in the risk of hypertension in participants with TPA in second quantile was not statistically significant before and after adjustment (P > 0.05). In addition, an inverse association between the TPA level and the risk of hypertension was observed in all three models (P for trend < 0.001).

**Table 2:** Logistic regression analysis for the associations between TPA and the risk of hypertension.

**TPA**	***N***	**Univariate**		**Model 1**		**Model 2**	
		**OR (95% CI)**	***P*** **-value**	**OR (95% CI)**	***P*** **-value**	**OR (95% CI)**	***P*** **-value**
**Q1**	2,591	1		1		1	
**Q2**	4,542	0.91 (0.79, 1.03)	0.136	0.95 (0.83, 1.08)	0.421	0.96 (0.84, 1.09)	0.526
**Q3**	3,564	0.75 (0.65, 0.86)	< 0.001	0.82 (0.71, 0.94)	0.004	0.82 (0.72, 0.95)	0.007
**Q4**	3,569	0.69 (0.60, 0.79)	< 0.001	0.77 (0.67, 0.89)	< 0.001	0.78 (0.68, 0.91)	0.001
**P for trend**		< 0.001		< 0.001		0.001	

OR, odds ratio; CI, confidence interval; Model 1 was adjusted for age, sex, education, marital status, and residency; Model 2 was further adjusted for smoking status, drinking frequency, sleep duration and annual income.

#### Frequency of PA and the risk of hypertension

Table 3 indicates the binary logistic regression results for the associations between PA frequency and the risk of hypertension. Compared with inactivity, there was an association between taking part in VPA 1–2 d/w (OR 0.80, 95%CI 0.65–0.99) and the risk of hypertension, but the association became insignificant after adjustment for confounders (Model 1: OR 0.85, 95%CI 0.69 to 1.06; Model 2: OR 0.86, 95%CI 0.69 to 1.07). Taking part in VPA 6–7 d/w (OR 0.77, 95%CI 0.68–0.87) was associated with a lower risk of hypertension, and the association remained statistically significant after adjusting for confounders (Model 1: OR 0.81, 95%CI 0.72–0.92; Model 2: OR 0.82, 95%CI 0.73–0.93). Taking part in MPA 6–7 d/w (OR 0.81, 95%CI 0.73–0.89) was associated with a lower risk of hypertension. After adjustment for confounders, the association attenuated slightly but remained significant (Model 1: OR 0.89, 95%CI 0.80–0.99; Model 2: OR 0.89, 95%CI 0.80–0.99). No association between any LPA frequency and hypertension was observed before and after adjusting.

**Table 3:** Logistic regression analysis for the associations between PA and the risk of hypertension.

**Variables**	***N***	**Univariate**		**Model 1**		**Model 2**	
		**OR (95% CI)**	***P*** **-value**	**OR (95% CI)**	***P*** **-value**	**OR (95% CI)**	***P*** **-value**
**Frequency**							
**VPA**							
No activity	9,374	1		1		1	
1–2 d/w	778	0.80 (0.65, 0.99)	0.043	0.85 (0.69, 1.06)	0.153	0.86 (0.69, 1.07)	0.178
3–5 d/w	1,144	0.95 (0.79, 1.12)	0.517	1.01 (0.85, 1.20)	0.902	1.02 (0.86, 1.21)	0.819
6–7 d/w	2,970	0.77 (0.68, 0.87)	< 0.001	0.81 (0.72, 0.92)	0.001	0.82 (0.73, 0.93)	0.003
**MPA**							
No activity	7,081	1		1		1	
1–2 d/w	945	0.93 (0.77, 1.12)	0.417	0.99 (0.82, 1.19)	0.902	0.99 (0.82, 1.19)	0.885
3–5 d/w	1,460	0.88 (0.75, 1.03)	0.117	0.96 (0.82, 1.12)	0.609	0.96 (0.82, 1.13)	0.619
6–7 d/w	4,780	0.81 (0.73, 0.89)	< 0.001	0.89 (0.80, 0.99)	0.034	0.89 (0.80, 0.99)	0.038
**LPA**							
No activity	2,380	1		1		1	
1–2 d/w	483	0.79 (0.59, 1.06)	0.117	0.83 (0.63, 1.11)	0.214	0.85 (0.64, 1.13)	0.257
3–5 d/w	1,141	1.06 (0.87, 1.28)	0.581	1.15 (0.95, 1.39)	0.162	1.17 (0.96, 1.41)	0.117
6–7 d/w	10,262	0.92 (0.81, 1.04)	0.168	0.96 (0.85, 1.09)	0.560	0.98 (0.87, 1.11)	0.744
**Duration**							
**VPA**							
No activity	9,347	1		1		1	
10–29 min/d	139	0.77 (0.47, 1.27)	0.132	0.83 (0.50, 1.37)	0.462	0.84 (0.51, 1.39)	0.509
30–119 min/d	905	0.82 (0.67, 0.99)	0.046	0.87 (0.71, 1.06)	0.176	0.88 (0.72, 1.07)	0.209
120–239 min/d	1,074	0.89 (0.75, 1.07)	0.230	0.93 (0.77, 1.11)	0.400	0.93 (0.77, 1.11)	0.419
≥240 min/d	2,774	0.79 (0.69, 0.89)	< 0.001	0.84 (0.74, 0.96)	0.009	0.85 (0.75, 0.97)	0.016
**MPA**							
No activity	7,081	1		1		1	
10–29 min/d	824	0.84 (0.69, 1.04)	0.107	0.90 (0.73, 1.11)	0.328	0.91 (0.77, 1.12)	0.385
30–119 min/d	2,910	0.90 (0.79, 1.01)	0.083	0.99 (0.87, 1.11)	0.822	0.98 (0.87, 1.11)	0.803
120–239 min/d	1,655	0.84 (0.73, 0.98)	0.029	0.92 (0.79, 1.07)	0.257	0.91 (0.78, 1.06)	0.238
≥240 min/d	1,796	0.74 (0.63, 0.86)	< 0.001	0.82 (0.71, 0.96)	0.014	0.83 (0.71, 0.97)	0.017
**LPA**							
No activity	2,380	1		1		1	
10–29 min/d	1,353	1.09 (0.92, 1.31)	0.313	1.13 (0.95, 1.36)	0.168	1.15 (0.96, 1.37)	0.138
30–119 min/d	5,768	0.95 (0.83, 1.08)	0.442	0.99 (0.87, 1.14)	0.951	1.01 (0.89, 1.16)	0.849
120–239 min/d	2,658	0.87 (0.75, 1.01)	0.076	0.90 (0.77, 1.06)	0.201	0.92 (0.78, 1.07)	0.274
≥240 min/d	2,107	0.83 (0.70, 0.97)	0.023	0.91 (0.77, 1.07)	0.258	0.92 (0.78, 1.09)	0.363

OR, odds ratio; CI, confidence interval; Model 1 was adjusted for age, sex, education, marital status, and residency; Model 2 was further adjusted for smoking status, drinking frequency, sleep duration, annual income.

#### Duration of PA and the risk of hypertension

Lower risk of hypertension was observed in participants spending 30–119 min (OR 0.82, 95%CI 0.67–0.99) or over 240 min per day (OR 0.79, 95%CI 0.69–0.89) for VPA. Spending 120–239 min (OR 0.84, 95%CI 0.73–0.98) or over 240 min (OR 0.74, 95%CI 0.63–0.86) on MPA each time was correlated with smaller odds of hypertension. Participants performing over 240 min LPA each time had a lower risk of hypertension (OR 0.83, 95%CI 0.70–0.97). After adjusting for age, sex, marital status, education, residency, smoking status, drinking frequency and sleep duration, spending over 240 min on VPA each time and over 240 min on MPA each time was correlated with smaller odds of hypertension (Table 3).

#### Dose-response relationship between TPA and hypertension

**Figure 1:**
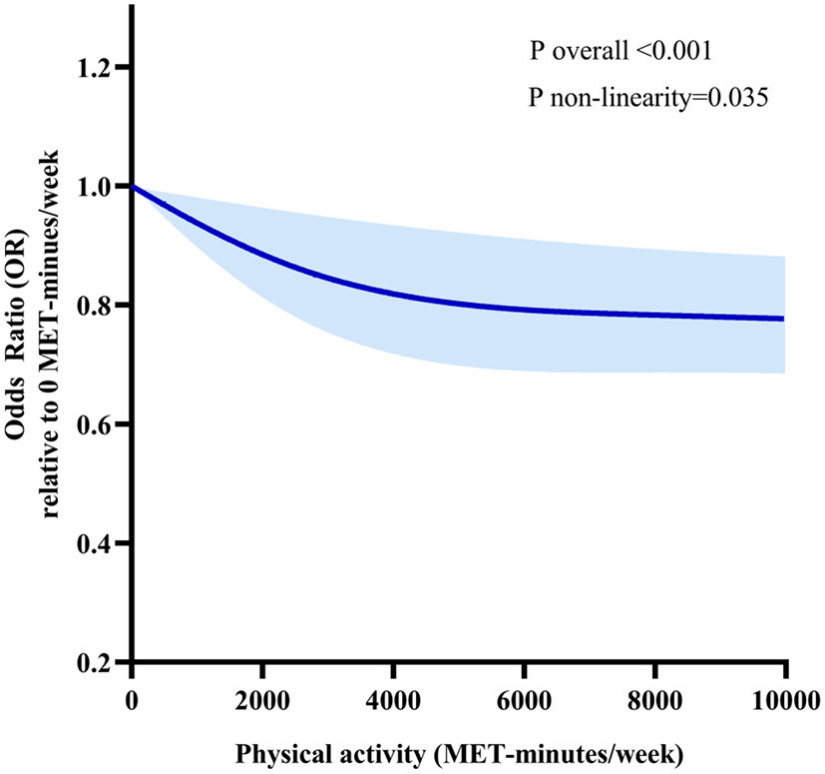
Dose-response relationships between TPA and the risk of hypertension. Models are adjusted for age, sex, marital status, education, residency, smoking status, drinking frequency, sleep duration and annual income. The solid line and long dash line represent the estimated odds ratio and its 95% confidence interval. Knots are at the 25^th^, 50^th^ and 75^th^ percentiles for TPA.

Figure 1 displays the RCS regression analysis result. We observed a non-linear dose-response association between TPA and the risk of hypertension among all of the participants (P non-linearity < 0.05). As the increase of TPA per week, the OR of hypertension markedly decreased. The point estimate of the risk ratio reached 0.85 (95% CI = 0.75–0.95) for activity at 3000 MET-minutes per week, indicating that the probability of having hypertension was 15% lower at that level of physical activity. When TPA reached 4500 MET-minutes per week, the risk of hypertension decreased by 19% (OR 0.81, 95%CI 0.70–0.93). The risk of hypertension no longer decreased significantly when the TPA was increased further.

### Subgroup and sensitivity analysis

As shown in Supplementary Figure S1, after sex stratification, there was a linear dose-response association between TPA and the risk of hypertension in male participants (P non-linearity > 0.05). The OR of hypertension decreased sharply as the TPA per week increased. However, no significant relationship was found in female participants. In the age-stratified analyses (Supplementary Figure S2), a nonlinear dose-response relationship was observed in those aged 45–64 (P non-linearity < 0.05). A linear dose-response relationship was observed in those aged ≥ 65 (P non-linearity > 0.05). All of the dose-response relationships revealed an inverse relationship between TPA and the risk of hypertension.

Missing values were imputed by multiple imputation, 19,466 participants were included in the sensitivity analysis. A non-linear dose-response relationship between TPA and the risk of hypertension was similar to the main analysis (Supplementary Figure S3).

## Discussion

This cross-sectional study evaluated the association between the risk of hypertension with various dimensions of PA in middle and older-aged Chinese people. Several important findings of the current study include: (1) Higher frequency and longer duration of VPA or MPA were significantly associated with a lower risk of hypertension. (2) There was an inverse association between TPA and risk for hypertension. (3) A non-linear dose-response relationship between TPA and the risk of hypertension was observed in all participants, the downward hypertension risk flattened when TPA was approximately 4500 MET-minutes/week. (4) There were sex and age differences in the dose-response relationship between physical activity and hypertension risk.

Intensity, frequency, and duration of PA are important factors affecting the benefits of PA. We found that VPA and MPA were associated with a lower risk of hypertension after adjustment for covariates. The result is consistent with those of previous studies, moderate-to-vigorous PA is beneficially associated with many health statuses (23). However, the association between moderate-to-vigorous PA with hypertension risk was only observed at 6–7days per week and duration ≥240 min. A single session of exercise can cause a sustained drop in blood pressure, a phenomenon known as post-exercise hypotension (24). Repeated induction of post-exercise hypotension at a higher frequency may be best in hypertensive patients. Sharman et al. (25) support the idea that hypertensive patients should be physically active most days of the week for optimal health benefits. The guideline (26) also encourage adults with high blood pressure to do moderate-to-vigorous aerobic exercise most days of the week, and preferably all days.

In terms of duration, the physical activity guidelines recommend that adults should do at least 150–300 min MPA per week, or 75–150 min. VPA per week for substantial health benefits (27). Taylor-Tolbert et al. (28) found that just 45 min of acute exercise immediately reduced blood pressure load in elderly male hypertensive patients. Our findings differ significantly from the previous reports. Smith et al. (29) compared self-reported and objectively measured physical activity trajectories in prostate cancer survivors, and they found that self-reported measures greatly overestimated moderate to vigorous physical activity. However, a systematic review noted that self-reported measures of physical activity were higher or lower than directly measured physical activity levels, depending on the measure of physical activity employed, the level of physical activity measured, and the gender of participants (30). The physical activity indicators associated with lowering hypertension in our results still need to be further validated with objective monitoring data. No significant association was observed in any frequency and duration of LPA after adjustment for confounders. Despite the evidence that even LPA is associated with numerous positive health outcomes (31), it remains unclear whether LPA can have a beneficial effect on hypertension.

Our result shows an inverse association between TPA level and the risk of hypertension, which is consistent with previous research (32, 33). Importantly, the effect of PA on hypertension is dose-dependent (16). We used RCS to explore this relationship and found the relationship to be non-linear, which decreases initially and then stabilizes. When TPA reached 3000 MET-minutes per week, the risk of hypertension decreased by 15%. The energy expenditure far exceeds the recommendations of current physical activity guidelines, suggestive that exceeding current guidelines in terms of energy expenditure incurs greater contribution. But the curve reached a plateau when TPA per week was approximately 4500 MET-mins. This is probably a volume for optimal health benefits, equivalent to 2.5 h a day of moderate-intensity, or 1.3 h a day of vigorous-intensity physical activity. A beneficial effect of physical exercise on hypertension appears in the regulation of endothelial function (34). Physical exercise augments blood flow and laminar shear stress, and it can also elicit systemic molecular pathways connected with angiogenesis and chronic anti-inflammatory action (35).

It is worth noting that sex and age differences were found in the dose-response relationship between physical activity and hypertension risk. A linear dose-response relationship between PA and hypertension was observed in the male population. No significant dose-response relationship was found in female participants, which was consistent with the findings of Haapanen et al. (17). Physical activity may differ between men and women. A previous report on the physical activity of middle and older Chinese indicated that men were more active in VPA and women were more active in MPA (36). In addition, the dose-response relationship between physical activity and the risk of hypertension in aged 45–64 participants is linear, but it was non-linear in those aged ≥65 participants. The age difference in the relation between PA and hypertensive risk may result from an age-related increase in arterial stiffness (18). Casey et al. (37) found that the vasodilator responses were slower in older adults at all exercise intensities compared with younger adults. The decreasing physical function makes it difficult for older adults to engage in moderate-to-vigorous PA (38), which also limits the higher benefits they can get from PA.

This study has the strength of including a representative sample of middle-aged and older adults in the Chinese population, which increased the statistical power. We conducted subgroup analyses based on age and sex to explore whether a difference in association could be observed in subgroups with a different age or sex. Furthermore, the sensitivity analyses have confirmed the robustness of this study's findings. This study has some limitations. First, because of the cross-sectional design, the cause-effect relationships between PA and the risk of hypertension not be determined. Further longitudinal studies are needed to determine the direction of causality for these associations. Second, questionnaire-based assessments of physical activity may be subject to recall and social desirability bias. Although the IPAQ is widely used, validation studies have shown poor agreement with objective measures (39). Third, these data do not allow us to distinguish the source of physical activity, it's hard to determine if the observed effects are independent of the type of occupation. Moreover, we were unable to include some important covariates, such as BMI, history of metabolic conditions, salt intake, medication use, and other potential confounders that may interfere with the effect of physical activity on hypertension risk. Our results cannot be excluded from being influenced by unknown or unmeasured confounders, despite the multivariable adjustments we made. Finally, results are limited to Chinese middle-aged and elderly groups, and may not generalize to other countries or populations.

## Implications

It is expected that the prevalence of hypertension will continually increase in the future as the Chinese population ages rapidly. Our findings reinforce a growing body of independent evidence that supports the effectiveness of moderate-to-vigorous-intensity physical activity in preventing and managing the risk of hypertension. This study also has significant practical implications. We analyzed the associations between multiple dimensions of physical activity and the risk of hypertension, and found gender and age differences in the dose-response relationship between accumulated physical activity over the week and the risk of hypertension. This may provide more detailed guidance for the prevention and control of hypertension in different populations. Physical activity as a non-pharmacological intervention has been shown to be effective in reducing the risk of hypertension. Objective measurement of physical activity should be considered and incorporated into the management of hypertensive patients.

## Conclusion

The results of this study assessed associations between different dimensions of PA and the risk of hypertension. After adjustment for potential confounders, higher frequency and longer duration of VPA or MPA were significantly associated with a lower risk of hypertension. Higher PA levels were associated with a lower risk of hypertension and there was an inverse dose-response relationship between weekly TPA and the risk of hypertension. When TPA reached approximately 4500 MET-minutes per week, the risk of hypertension in the overall population decreased by 19%, while continued increases in PA did not appear to produce any further marked reduction in the risk. These findings provide further proof that hypertension could be prevented through increased PA.

## Data availability statement

Publicly available datasets were analyzed in this study. This data can be found at: https://g2aging.org.

## Ethics statement

The studies involving human participants were reviewed and approved by the Biomedical Ethics Review Committee of Peking University. The patients/participants provided their written informed consent to participate in this study.

## Author contributions

LZ, YL, and WF designed the research. LZ, WF, NX, XY, and MW performed all the statistical analysis. LZ, WF, YL, and XW interpreted the data analysis. LZ, WF, YC, YL, and XW wrote the manuscript with critical input from YL. All authors read and approved the final manuscript.

## Funding

This work was supported by the Jiangsu Provincial Natural Science Foundation of China (grant number, BK20190999), the Xuzhou Natural Science Foundation (grant number, KC21029), and the Natural Science Foundation of the Jiangsu Education Institutions of China (grant number, 22KJB180017).

## Conflict of interest

The authors declare that the research was conducted in the absence of any commercial or financial relationships that could be construed as a potential conflict of interest.

## Publisher's note

All claims expressed in this article are solely those of the authors and do not necessarily represent those of their affiliated organizations, or those of the publisher, the editors and the reviewers. Any product that may be evaluated in this article, or claim that may be made by its manufacturer, is not guaranteed or endorsed by the publisher.

## Supplementary material

Supplementary Figure S1 Dose-response relationships between TPA and the risk of hypertension for different sex groups (A) Males. (B) Females. Models were adjusted for age, marital status, education, residency, smoking status, drinking frequency, sleep duration and annual income. The solid line and long dash line represent the estimated odds ratio and its 95% confidence interval. Knots are at the 25th, 50th, and 75th percentiles for TPA.

Supplementary Figure S2 Dose-response relationships between TPA and the risk of hypertension for different age groups (A) Participants aged < 65 years. (B) Participants aged 65 years and older. Models were adjusted for sex, age, marital status, education, residency, smoking status, drinking frequency, sleep duration and annual income. The solid line and long dash line represent the estimated odds ratio and its 95% confidence interval. Knots are at the 25th, 50th and 75th percentiles for TPA.

Supplementary Figure S3 Sensitivity analyses of dose-response relationships between TPA and the risk of hypertension. Models are adjusted for age, sex, marital status, education, residency, smoking status, drinking frequency, sleep duration and annual income. The solid line and long dash line represent the estimated odds ratio and its 95% confidence interval. Knots are at the 25th, 50th and 75th percentiles for TPA.

Supplementary Table S1 Physical activity level of participants.

## Abbreviations

PA: physical activity
CHARLS: China Health and Retirement Longitudinal Study
RCS: restricted cubic spline
VPA: vigorous physical activity
MPA: moderate physical activity
LPA: light physical activity
TPA: total physical activity
MET: Metabolic equivalent of task
OR: Odds ratio
Cis: confidence intervals.

## References

[1] Kearney PM Whelton M Reynolds K Muntner P Whelton PK He J. Global burden of hypertension: analysis of worldwide data. Lancet. (2005) 365:217–23. 10.1016/S0140-6736(05)17741-1 15652604

[2] Staessen JA Wang J Bianchi G Birkenhäger WH. Essential hypertension. Lancet. (2003) 361:1629–41. 10.1016/S0140-6736(03)13302-8 12747893

[3] Williams B Mancia G Spiering W Agabiti RE Azizi M Burnier M. 2018 esc/esh guidelines for the management of arterial hypertension: the task force for the management of arterial hypertension of the european society of cardiology and the european society of hypertension: the task force for the management of arterial hypertension of the european society of cardiology and the european society of hypertension. J Hypertens. (2018) 36:1953–2041. 10.1097/HJH.0000000000001940 30234752

[4] Whelton PK Carey RM Aronow WS Casey DJ Collins KJ Dennison HC. 2017 acc/aha/aapa/abc/acpm/ags/apha/ash/aspc/nma/pcna guideline for the prevention, detection, evaluation, and management of high blood pressure in adults: a report of the american college of cardiology/american heart association task force on clinical practice guidelines. Hypertension. (2018) 71:e13–115. 10.1161/HYP.0000000000000076 29133356

[5] Kuo PL Pu C. The contribution of depression to mortality among elderly with self-reported hypertension: analysis using a national representative longitudinal survey. J Hypertens. (2011) 29:2084–90. 10.1097/HJH.0b013e32834b59ad 21934532

[6] Li Y Yang L Wang L Zhang M Huang Z Deng Q. Burden of hypertension in china: a nationally representative survey of 174,621 adults. Int J Cardiol. (2017) 227:516–23. 10.1016/j.ijcard.2016.10.110 27856040

[7] Hall ME Do CJ Da SA Juncos LA Wang Z Hall JE. Obesity, hypertension, and chronic kidney disease. Int J Nephrol Renovasc Dis. (2014) 7:75–88. 10.2147/IJNRD.S39739 24600241 PMC3933708

[8] Shimbo D Levitan EB Booth JR Calhoun DA Judd SE Lackland DT. The contributions of unhealthy lifestyle factors to apparent resistant hypertension: findings from the reasons for geographic and racial differences in stroke (regards) study. J Hypertens. (2013) 31:370–6. 10.1097/HJH.0b013e32835b6be7 23303356 PMC3838894

[9] Batis C Gordon-Larsen P Cole SR Du S Zhang B Popkin B. Sodium intake from various time frames and incident hypertension among Chinese adults. Epidemiology. (2013) 24:410–8. 10.1097/EDE.0b013e318289e047 23466527 PMC3909658

[10] O'Donovan C Lithander FE Raftery T Gormley J Mahmud A Hussey J. Inverse relationship between physical activity and arterial stiffness in adults with hypertension. J Phys Act Health. (2014) 11:272–7. 10.1123/jpah.2012-0075 23359316

[11] Li X Liu Q Wang K Li R Hou Y Li Z. Prevalence and correlates of physical inactivity among elderly Chinese. Osteoarthr Cartilage. (2017) 25:S203. 10.1016/j.joca.2017.02.349

[12] Cheng YL Leung CB Kwan TH Chau KF Tong KL Li CS. Screening for high blood pressure (bp): the Hong Kong experience. Int J Cardiol. (2009) 137:S126. 10.1016/j.ijcard.2009.09.429

[13] Treff C Benseñor IM Lotufo PA. Leisure-time and commuting physical activity and high blood pressure: the Brazilian longitudinal study of adult health (elsa-brasil). J Hum Hypertens. (2017) 31:278–83. 10.1038/jhh.2016.75 27734826

[14] Chase NL Sui X Lee DC Blair SN. The association of cardiorespiratory fitness and physical activity with incidence of hypertension in men. Am J Hypertens. (2009) 22:417–24. 10.1038/ajh.2009.6 19197248

[15] Williams PT. A cohort study of incident hypertension in relation to changes in vigorous physical activity in men and women. J Hypertens. (2008) 26:1085–93. 10.1097/HJH.0b013e3282fb81dc 18475145 PMC2828465

[16] Pescatello LS Buchner DM Jakicic JM Powell KE Kraus WE Bloodgood B. Physical activity to prevent and treat hypertension: a systematic review. Med Sci Sports Exerc. (2019) 51:1314–23. 10.1249/MSS.0000000000001943 31095088

[17] Haapanen N Miilunpalo S Vuori I Oja P Pasanen M. Association of leisure time physical activity with the risk of coronary heart disease, hypertension and diabetes in middle-aged men and women. Int J Epidemiol. (1997) 26:739–47. 10.1093/ije/26.4.739 9279605

[18] Cohen L Curhan GC Forman JP. Influence of age on the association between lifestyle factors and risk of hypertension. J Am Soc Hypertens. (2012) 6:284–90. 10.1016/j.jash.2012.06.002 22789880 PMC3721664

[19] You Y Teng W Wang J Ma G Ma A Wang J. Hypertension and physical activity in middle-aged and older adults in china. Sci Rep. (2018) 8:16098. 10.1038/s41598-018-34617-y 30382177 PMC6208349

[20] Pavey TG Peeters G Bauman AE Brown WJ. Does vigorous physical activity provide additional benefits beyond those of moderate? Med Sci Sports Exerc. (2013) 45:1948–55. 10.1249/MSS.0b013e3182940b91 23542895

[21] Craig CL Marshall AL Sjöström M Bauman AE Booth ML Ainsworth BE. International physical activity questionnaire: 12-country reliability and validity. Med Sci Sports Exerc. (2003) 35:1381–95. 10.1249/01.MSS.0000078924.61453.FB 12900694

[22] Physical AGAC. Physical Activity Guidelines Advisory Committee Report, 2008. Washington, DC: US Department of Health and Human Services (2008).

[23] Warburton DE Nicol CW Bredin SS. Health benefits of physical activity: the evidence. CMAJ. (2006) 174:801–9. 10.1503/cmaj.051351 16534088 PMC1402378

[24] Kenney MJ Seals DR. Postexercise hypotension. Key features, mechanisms, and clinical significance Hypertension. (1993) 22:653–64. 10.1161/01.HYP.22.5.653 8225525

[25] Sharman JE La Gerche A Coombes JS. Exercise and cardiovascular risk in patients with hypertension. Am J Hypertens. (2015) 28:147–58. 10.1093/ajh/hpu191 25305061

[26] Whelton PK Carey RM Aronow WS Casey DJ Collins KJ Dennison HC. 2017 acc/aha/aapa/abc/acpm/ags/apha/ash/aspc/nma/pcna guideline for the prevention, detection, evaluation, and management of high blood pressure in adults: a report of the american college of cardiology/american heart association task force on clinical practice guidelines. J Am Coll Cardiol. (2018) 71:e127–248. 10.1016/j.jacc.2017.11.006 29146535

[27] Piercy KL Troiano RP Ballard RM Carlson SA Fulton JE Galuska DA. The physical activity guidelines for americans. JAMA. (2018) 320:2020–8. 10.1001/jama.2018.14854 30418471 PMC9582631

[28] Taylor-Tolbert NS Dengel DR Brown MD McCole SD Pratley RE Ferrell RE. Ambulatory blood pressure after acute exercise in older men with essential hypertension. Am J Hypertens. (2000) 13:44–51. 10.1016/S0895-7061(99)00141-7 10678270

[29] Smith L Lee JA Mun J Pakpahan R Imm KR Izadi S. Levels and patterns of self-reported and objectively-measured free-living physical activity among prostate cancer survivors: a prospective cohort study. Cancer-Am Cancer Soc. (2019) 125:798–806. 10.1002/cncr.31857 30516839 PMC6378115

[30] Prince SA Adamo KB Hamel ME Hardt J Connor GS Tremblay MA. Comparison of direct versus self-report measures for assessing physical activity in adults: a systematic review. Int J Behav Nutr Phys Act. (2008) 5:56. 10.1186/1479-5868-5-56 18990237 PMC2588639

[31] Füzéki E Engeroff T Banzer W. Health benefits of light-intensity physical activity: a systematic review of accelerometer data of the national health and nutrition examination survey (nhanes). Sports Med. (2017) 47:1769–93. 10.1007/s40279-017-0724-0 28393328

[32] Bakker EA Sui X Brellenthin AG Lee DC. Physical activity and fitness for the prevention of hypertension. Curr Opin Cardiol. (2018) 33:394–401. 10.1097/HCO.0000000000000526 29762150

[33] Castro I Waclawovsky G Marcadenti A. Nutrition and physical activity on hypertension: implication of current evidence and guidelines. Curr Hypertens Rev. (2015) 11:91–9. 10.2174/1573402111666150429170302 25921545

[34] Gambardella J Morelli MB Wang XJ Santulli G. Pathophysiological mechanisms underlying the beneficial effects of physical activity in hypertension. J Clin Hypertens. (2020) 22:291–5. 10.1111/jch.13804 31955526 PMC7169989

[35] Di Francescomarino S Sciartilli A Di Valerio V Di Baldassarre A Gallina S. The effect of physical exercise on endothelial function. Sports Med. (2009) 39:797–812. 10.2165/11317750-000000000-00000 19757859

[36] Wang R Bishwajit G Zhou Y Wu X Feng D Tang S. Intensity, frequency, duration, and volume of physical activity and its association with risk of depression in middle- and older-aged chinese: evidence from the china health and retirement longitudinal study, 2015. PLoS ONE. (2019) 14:e221430. 10.1371/journal.pone.0221430 31425559 PMC6699736

[37] Casey DP Ranadive SM Joyner MJ. Aging is associated with altered vasodilator kinetics in dynamically contracting muscle: role of nitric oxide. J Appl Physiol. (1985) 119: 232–41. 10.1152/japplphysiol.00787.2014 26023230 PMC4526703

[38] Rolland Y Lauwers-Cances V Cristini C Abellan VKG Janssen I Morley JE. Difficulties with physical function associated with obesity, sarcopenia, and sarcopenic-obesity in community-dwelling elderly women: the epidos (epidemiologie de l'osteoporose) study. Am J Clin Nutr. (2009) 89:1895–900. 10.3945/ajcn.2008.26950 19369381

[39] Macfarlane DJ Lee CC Ho EY Chan KL Chan DT. Reliability and validity of the Chinese version of ipaq (short, last 7 days). J Sci Med Sport. (2007) 10:45–51. 10.1016/j.jsams.2006.05.003 16807105

