# Supplementary figures and images for: Association between physical activity dimensions and the risk of hypertension among middle and older adults: A cross-sectional study in China
Source: Front Public Health. 2022 Sep 24;10:995755. doi: 10.3389/fpubh.2022.995755 (PMC9547049; doi:10.3389/fpubh.2022.995755)

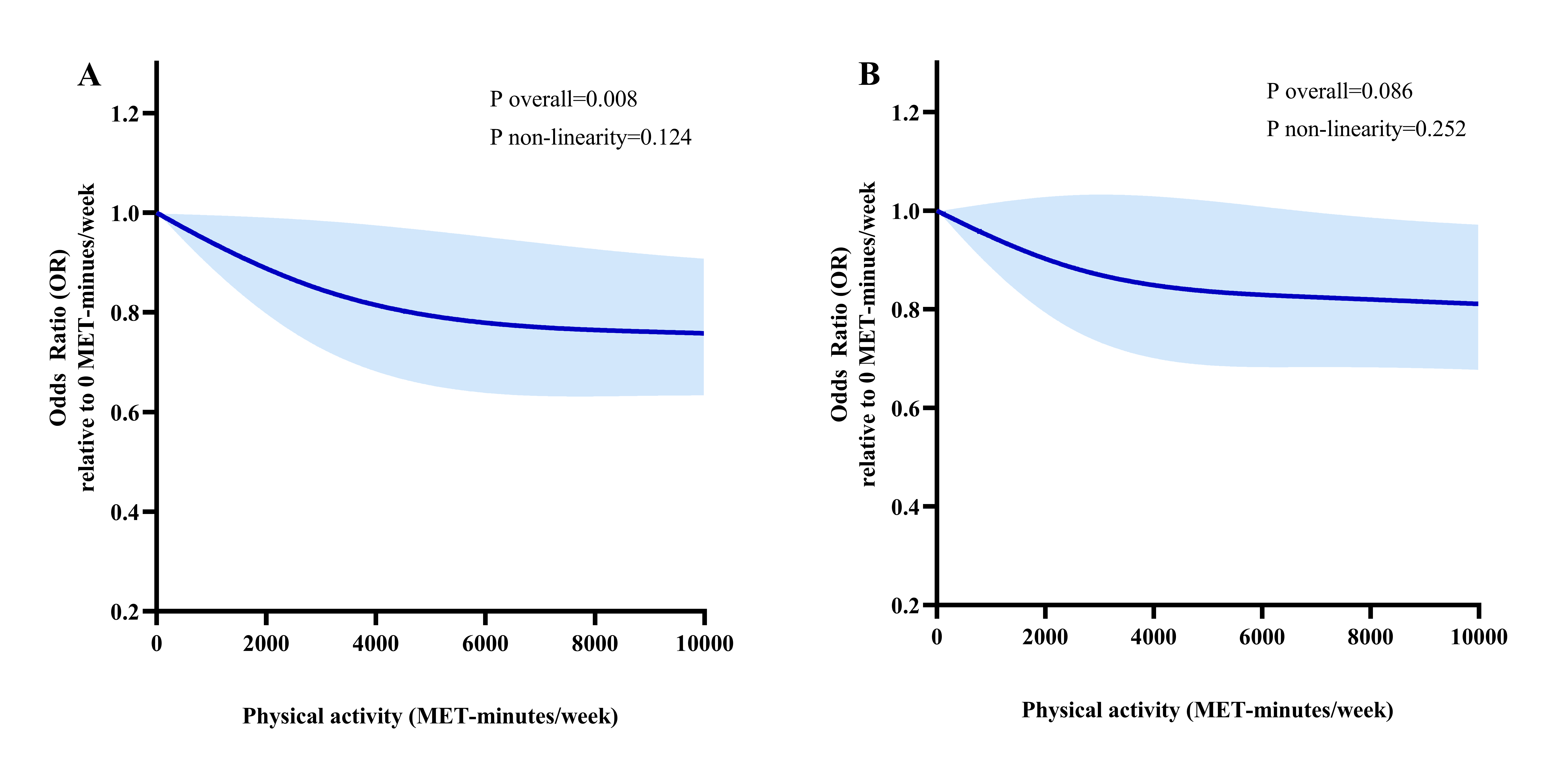

Supplement: Supplementary Figure S1 — Dose-response relationships between TPA and the risk of hypertension for different sex groups (A) Males. (B) Females. Models were adjusted for age, marital status, education, residency, smoking status, drinking frequency, sleep duration and annual income. The solid line and long dash line represent the estimated odds ratio and its 95% confidence interval. Knots are at the 25th, 50th, and 75th percentiles for TPA. [file Image_1.tif]

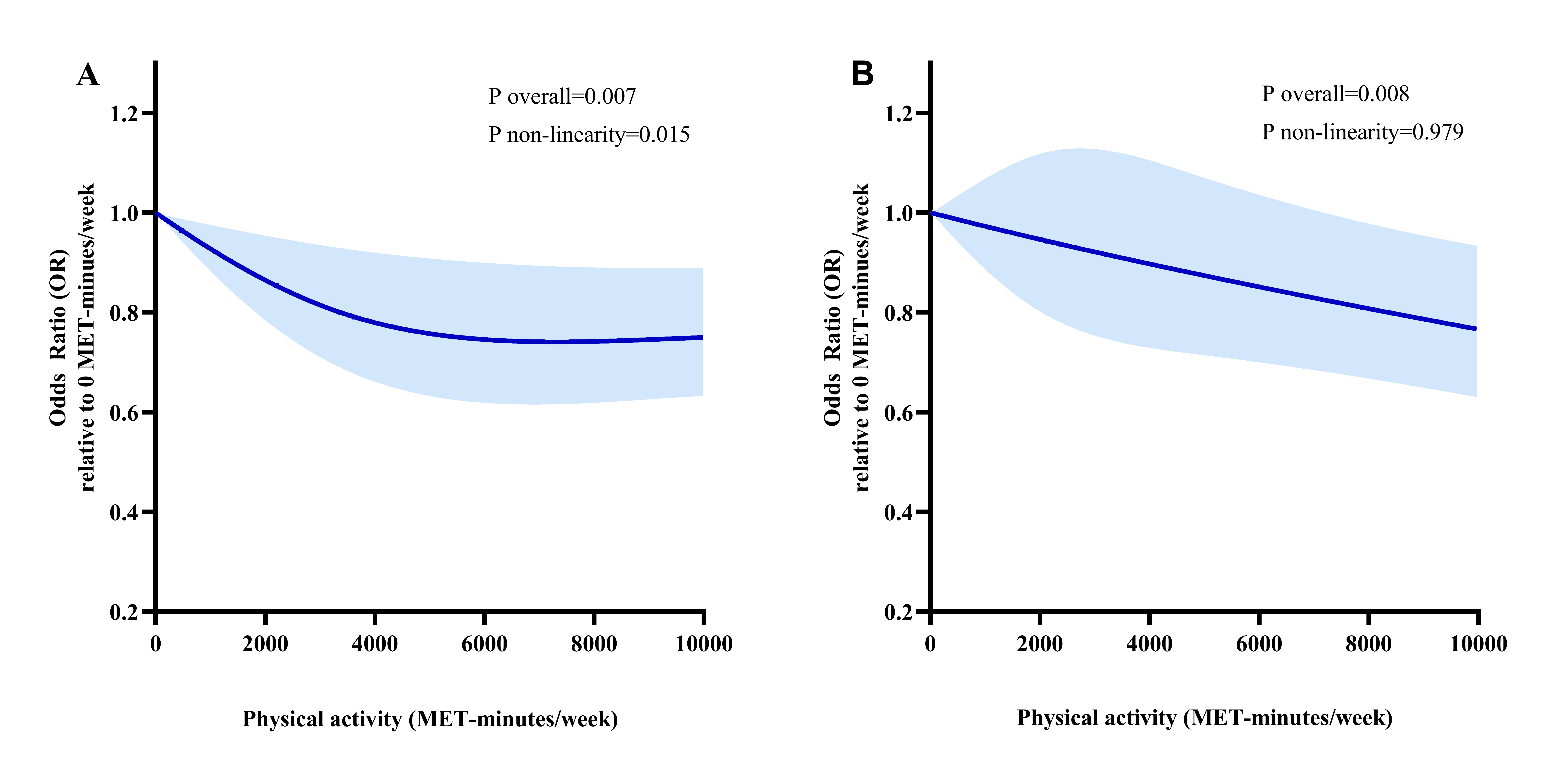

Supplement: Supplementary Figure S2 — Dose-response relationships between TPA and the risk of hypertension for different age groups (A) Participants aged < 65 years. (B) Participants aged 65 years and older. Models were adjusted for sex, age, marital status, education, residency, smoking status, drinking frequency, sleep duration and annual income. The solid line and long dash line represent the estimated odds ratio and its 95% confidence interval. Knots are at the 25th, 50th and 75th percentiles for TPA. [file Image_2.tif]

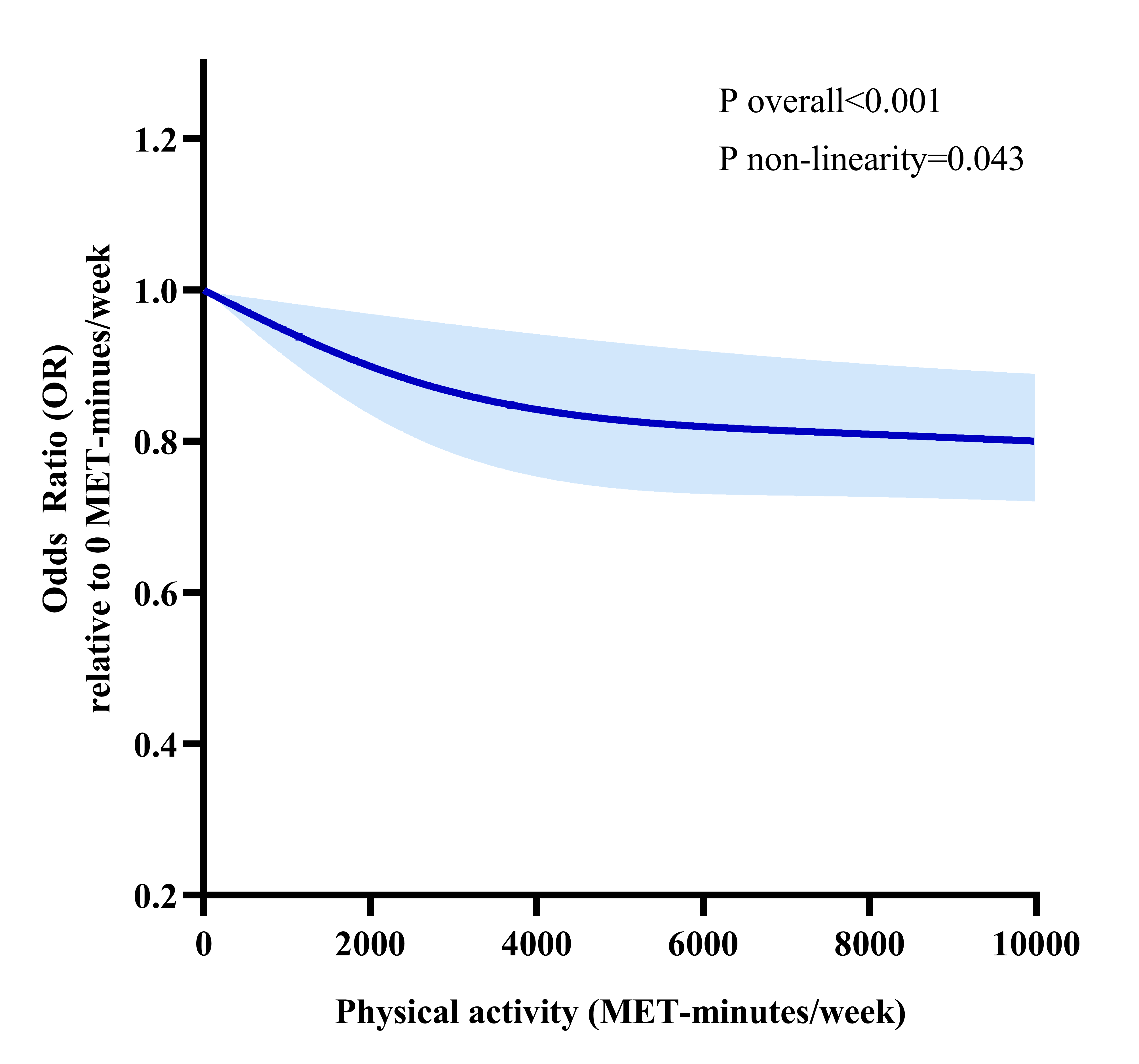

Supplement: Supplementary Figure S3 — Sensitivity analyses of dose-response relationships between TPA and the risk of hypertension. Models are adjusted for age, sex, marital status, education, residency, smoking status, drinking frequency, sleep duration and annual income. The solid line and long dash line represent the estimated odds ratio and its 95% confidence interval. Knots are at the 25th, 50th and 75th percentiles for TPA. [file Image_3.tif]
