# Supplementary material for: Association between physical activity dimensions and the risk of hypertension among middle and older adults: A cross-sectional study in China
Source: Front Public Health. 2022 Sep 24;10:995755. doi: 10.3389/fpubh.2022.995755 (PMC9547049; doi:10.3389/fpubh.2022.995755)
Supplement: Supplementary file 4 [file Table_1.pdf]

**The modified IPAQ-SF**

Now we would like to ask about the amount of time you spend on different types of physical activities in a usual week.

Now, think about all the vigorous activities requiring hard/high-intensity physical effort that you do in a usual week. Vigorous activities make you breathe much harder than normal and may include heavy lifting, digging, plowing, aerobics, fast bicycling, and cycling with a heavy load. Think only about those physical activities that you did for at least 10 minutes at a time.

1. During a usual week, did you do any vigorous activities for at least 10 minutes continuously?
   □ Yes ➔2
   □ No➔4
2. During a usual week, on how many days did you do vigorous activities for at least 10 minutes?
   └──┘ 1…7 days
3. How much time did you usually spend doing vigorous activities on one of those days?

| | |
|---|---|
| □< 2 hours | □< 30 minutes<br>□≥ 30 minutes |
| □≥ 2 hours | □< 4 hours<br>□≥ 4 hours |

Now think about activities which take moderate physical effort that you do in a usual week. Moderate physical activities make you breathe somewhat harder than normal and may include carrying light loads, bicycling at a regular pace, or mopping the floor. Again, think about only those physical activities that you did for at least 10 minutes at a time.

4. During a usual week, did you do any moderate activities for at least 10 minutes continuously?
   □ Yes ➔5
   □ No➔7
5. During a usual week, on how many days did you do moderate activities for at least 10 minutes?
   └──┘ 1…7 days
6. How much time did you usually spend doing moderate activities on one of those days?

| | |
|---|---|
| □< 2 hours | □< 30 minutes<br>□≥ 30 minutes |
| □≥ 2 hours | □< 4 hours<br>□≥ 4 hours |

Now think about the time you spend walking in a usual week. This includes at work and at home, walking to travel from place to place, and any other walking that you might do solely for recreation, sport, exercise, or leisure.

7. During a usual week, did you do any walking for at least 10 minutes continuously?
   □ Yes ➔8
   □ No
8. During a usual week, on how many days did you do walking for at least 10 minutes?

└──┘ 1…7 days

9. How much time did you usually spend doing walking on one of those days?

| | |
|---|---|
| □< 2 hours | □< 30 minutes<br>□≥ 30 minutes |
| □≥ 2 hours | □< 4 hours<br>□≥ 4 hours |
