# Supplementary material for: Association between physical activity dimensions and the risk of hypertension among middle and older adults: A cross-sectional study in China
Source: Front Public Health. 2022 Sep 24;10:995755. doi: 10.3389/fpubh.2022.995755 (PMC9547049; doi:10.3389/fpubh.2022.995755)
Supplement: Supplementary Table S1 — Physical activity level of participants. [file Table_2.pdf]

Table S1 Physical activity level of participants

Participants	N	Mean±SD	T	P-value
**Sex**			7.91	<0.001
Male	6903	5506.39±5863.02		
Female	7363	4762.42±5376.86		
**Age**			19.42	<0.001
<65	9483	5764.20±5853.16		
≥65	4783	3849.96±4916.07		
**Overall**	14266	5122.41±5629.45		
